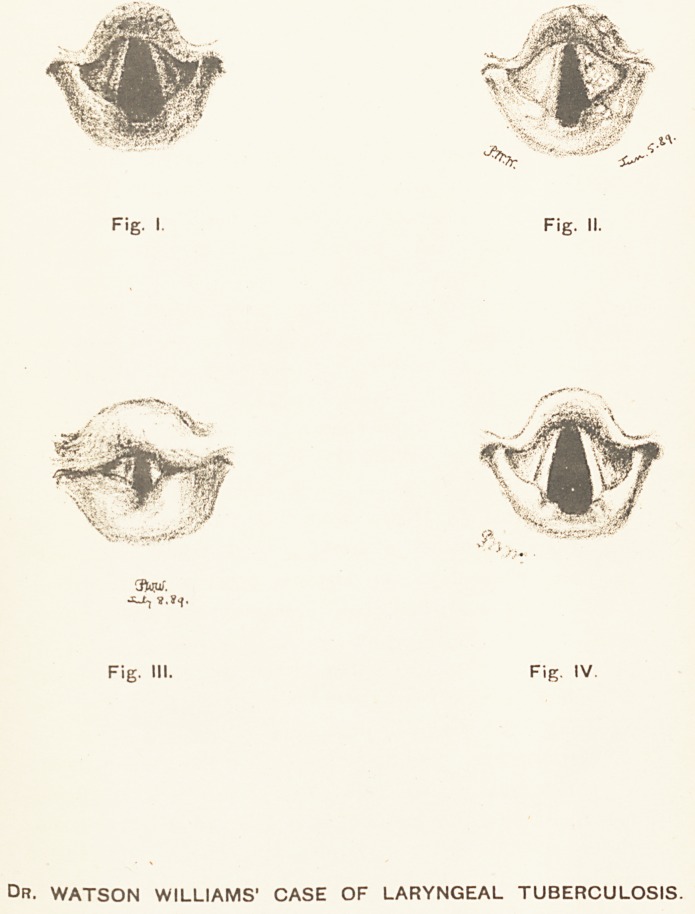# A Case of Laryngeal Tuberculosis

**Published:** 1891-03

**Authors:** P. Watson Williams

**Affiliations:** Physician in Charge of the Throat Department of the Bristol Royal Infirmary


### A CASE OF LARYNGEAL TUBERCULOSIS.

BY

P. WATSON WILLIAMS, M.B. Lond.,

*Physician in charge of the Throat Department of the Bristol Royal Infirmary.*

---

ON the 15th of May, 1889, I was asked to see Mrs. H., in consultation with her husband, a medical man, on account of frequent attacks of spasm of the glottis, giving rise to alarming dyspnœa. For five weeks previously she had had constant cough, marked salivation, severe pain in the pharynx during rest, but more severe on swallowing. The cough was worse on lying down at night, and frequently ended in vomiting, causing some loss of flesh. The soft palate was relaxed and hyperæmic, the uvula elongated without hypertrophy. The larynx was extremely hyperæmic (see Fig. I.), and there was slight general swelling of the mucous membrane. There was expectoration of clear bronchial mucus, and a few râles could be heard over the back and front of the chest. The right apex was somewhat dull on percussion, with expiratory breath-sounds prolonged and harsh. She was seven months pregnant. There was a family history of tuberculosis.

The elongated uvula was evidently the cause of the laryngeal spasms, and sufficiently accounted for all the symptoms and physical signs, except, perhaps, the well-marked salivation, which I attributed to her pregnant condition.

The elongated portion of the uvula was removed, the usual directions with regard to iced and fluid food being given, and we ordered a tonic.

As Mrs. H. resided in the country, about fifty miles by rail from Bristol, I did not see her again till May 22nd, when I found she had been greatly relieved. The attacks of dyspnœa and vomiting had not once recurred, and the pain was very much less severe. The larynx, however, was as red and injected as on my first interview.

I next saw her on June 5th. The pain on swallowing was as bad as ever, although the vomiting and glottic spasms had not recurred.

Examination of the larynx showed some thickening of the left margin of the epiglottis, also of the left ary-epiglottic fold and left false cord, and in these positions a few isolated tubercles had appeared. (See Fig. II.) The vocal cords were movable on phonation; the voice husky. She had a rapid, feeble pulse, and a hectic temperature chart. I had no hesitation in diagnosing laryngeal tuberculosis, and had the painful duty of conveying to the husband my opinion of the extreme gravity of the case, and advised the induction of premature labour at the eighth month. The frequent use of a local application by spray and brush of a solution of biniodide of mercury, 1 in 1000, was ordered, and the administration of creasote and cod-liver oil internally.

On July 8th, the larynx was typically tubercular (see Fig. III.), the epiglottis much thickened and pale, the right and left ary-epiglottic folds characteristically pale, tumid, and pear-shaped, the isolated tubercles having coalesced; no ulceration. There was also a small tubercle in the left anterior pillar of the fauces. The voice had become quite aphonic, and talking as well as swallowing was painful. The child had been born ten days. At the husband's request, I asked Mr. Mark Hovell to come down and see the patient. He confirmed my diagnosis, and pronounced the case to be practically hopeless, but suggested the use of a spray of 20 per cent. of lactic acid, to be gradually increased in strength. This was used once or twice, but induced so much vomiting and glottic spasm that, after reducing the strength of the lactic acid to 10 per cent. with similar results, the husband gave it up, and reverted to the biniodide of mercury treatment.

July 24th. As the use of lactic acid had proved so impossible, I increased the strength of the biniodide of mercury to 1 in 250, and advised inhalation of conium and creasote. By this time the tubercular deposits in the left ary-epiglottic fold had ulcerated. The patient was rapidly losing flesh, the expectoration was muco-purulent, and numerous bacilli were present.

The husband, in despair, soon after this took his wife to Sir Morell Mackenzie, hoping that there might be some error in diagnosis; but his opinion only confirmed the gloomy outlook. Returning home, the biniodide spray and local applications with the brush were steadily continued, in combination with various tonics.

I saw the patient from time to time, at intervals of about a month, and in November and December there was evidence of improvement in the condition of the larynx. By January 24th, 1890, there was no pain in her throat at all, not even on swallowing. The cough had quite gone. The voice had quite returned, but was a little thick. The patient suffered from great mental depression, but had improved in general appearance very much. The right apex no longer dull on percussion; no harsh breathing, but expiration was somewhat prolonged. All the tubercular deposit had been absorbed; the ulceration had never been extensive—in fact, at any time was only observable in the left ary-epiglottic fold. The mucous membrane of the epiglottis and ary-epiglottic folds was still a little thickened and hyperæmic, but only to a very slight degree. The vocal cords normal in colour, and freely movable.

Within a month the larynx became perfectly normal (see Fig. IV.), and when I saw her nine months later, on October 10th, the larynx was still perfectly normal in every respect. The patient had been improving all round, and had gained flesh. She had so far recovered her strength in the earlier months of the year that she had herself nursed her husband through an attack of the Russian influenza, so prevalent at that time. The right apex of the lung never quite cleared up.

Recovery after the occurrence of such very extensive tubercular disease of the larynx is almost phenomenal, and exceeded our most sanguine expectations. When I told my friend Mr. Hovell of the happy result obtained he was much astonished, remarking that he had never seen such an advanced case of laryngeal tuberculosis recover.

The only local therapeutic treatment was the application of the 1 in 250 solution of biniodide of mercury, persisted in for months. The fact of the patient residing at such a distance from Bristol fortunately prevented my adopting more radical procedures; and Mr. Hovell's suggestions were doubtless limited for the same reason.

I have treated two other cases of advanced laryngeal tuberculosis by this method, and although it has relieved the conditions to a certain extent, neither of them recovered. But they were both very advanced before I saw them, and the lungs in these cases much diseased.

Fig. I.

Fig. II.

Fig. III.

Fig. IV.

DR. WATSON WILLIAMS' CASE OF LARYNGEAL TUBERCULOSIS.

## Figures and Tables

**Fig. I. Fig. II. Fig. III. Fig. IV. f1:**